# Development of well-being assessment criteria for pharmacists: a mixed-methods study in the Eastern Special Development Zone of Thailand

**DOI:** 10.1080/20523211.2025.2605388

**Published:** 2026-01-09

**Authors:** Pongsatean Luengalongkot, Pattrawadee Makmee, Chaiwat Daorueng

**Affiliations:** a Faculty of Political Science and Law, Burapha University, Bangsaen, Thailand; b Department of Research and Applied Psychology, Faculty of Education, Burapha University, Bangsaen, Thailand; c Department of Psychology and Cognitive Science, University of Trento, Italy and College of Research Methodology and Cognitive Science, Burapha University, Bangsaen, Thailand

**Keywords:** Physical well-being, occupational well-being, emotional and psychological well-being, social well-being, financial well-being, spiritual well-being

## Abstract

**Background:**

Pharmacists’ capacity to handle their workload and fulfil health service expectations affect their well-being. This study aimed to analyze and validate the second-order confirmatory factor analysis of pharmacists’ well-being in the Eastern Economic Corridor (EEC) of Thailand and establish criteria for assessing pharmacists’ well-being.

**Methods:**

A mixed-methods explanatory sequential approach was employed in two phases. Phase 1: Quantitative study involving data collection from 400 pharmacists using a structured questionnaire with second-order confirmatory factor analysis (CFA). Phase 2: Qualitative study including in-depth interviews with seven key informants to refine assessment criteria and interpret quantitative results.

**Results:**

The quantitative findings indicated that the second-order confirmatory factor analysis of pharmacists’ well-being exhibited a good fit with the empirical data. The highest loading was observed for physical well-being (PWb), followed by occupational well-being (OWb), emotional well-being (EWb), social well-being (SWb), financial well-being (FWb), and spiritual well-being (SpWb). The qualitative findings provided robust support for the validity of the proposed factor structure. The pharmacists highlighted the significance of PWb, followed by OWb. They stated that the implementation of stress management strategies facilitated EWb. SWb was defined as the support received from colleagues, FWb was associated with the need for security, and SpWb was linked to the pursuit of meaning in one’s work.

**Conclusion:**

These findings advocate for the establishment of a programme for promoting well-being among pharmacists that focuses on their physical health, workplace enhancements, and increased professional recognition. Pharmacists’ well-being in the EEC is influenced by various factors such as physical health, professional engagement, emotional resilience, organisational systems, and work context. These insights can guide policy development, human resource management, and organisational wellness initiatives that can enhance the quality of life of pharmacists.

## Background

Thailand’s transformation into a modern, knowledge-based economy has fundamentally reshaped the nation's healthcare landscape, creating both opportunities and challenges for healthcare professionals. Central to this transformation is the establishment of the Eastern Special Development Zone of Thailand or the Eastern Economic Corridor (EEC) in 2017, a strategic development encompassing Chonburi, Rayong, and Chachoengsao provinces that represents Thailand’s ambitious vision for economic modernisation (Muensriphum et al., 2021; Office of the Eastern Economic Corridor, 2024). This economic transition has generated unprecedented demands on healthcare organisations within the EEC region, compelling them to adapt to rapidly evolving market dynamics, heightened competitiveness, and increasingly complex service expectations. As a consequence, pharmacy services have emerged as a critical component of modern healthcare delivery, with pharmacists assuming expanded roles that extend far beyond traditional medication dispensing (Price et al., 2023).

The evolution of pharmaceutical practice reflects broader changes occurring throughout the healthcare sector. Modern pharmacists have transitioned from passive medication distributors to active healthcare providers responsible for pharmaceutical supply chain management, clinical consultation, medication therapy management, and advocacy for rational drug utilisation (Alzarea et al., 2022). This professional transformation, while expanding the scope and impact of pharmacy practice, has simultaneously intensified the demands placed upon individual practitioners. Research consistently demonstrates that pharmacists’ capacity to manage increased workloads while meeting elevated health service expectations directly influences their professional well-being, which in turn affects their ability to provide quality patient care (Diener et al., 2018). Contemporary understanding of well-being encompasses a multidimensional construct influenced by pleasant emotions, life satisfaction, and the dynamic alignment between individuals and their social environment (Lomas et al., 2022). Furthermore, well-being maintains intrinsic connections to life meaning and psychological development, with its realisation dependent upon the satisfaction of fundamental human needs (Delle Fave et al., 2011; Ott, 2020).

This study addresses a critical gap in healthcare workforce research by developing the first validated well-being assessment framework specifically designed for pharmacists in Thailand's rapidly developing Eastern Economic Corridor. The research represents a significant contribution to both academic literature and practical healthcare management, offering innovative approaches that extend beyond traditional well-being assessments to address the complex realities of modern pharmacy practice in emerging economies.

The methodological innovation of this study lies in its sophisticated mixed-methods explanatory sequential design that combines rigorous quantitative analysis with rich qualitative insights. By employing second-order confirmatory factor analysis alongside in-depth qualitative interviews, the research creates comprehensive assessment criteria that capture both the statistical relationships between well-being components and the lived experiences of pharmacy professionals. This dual approach ensures that the resulting framework is not only psychometrically sound but also practically relevant to the daily challenges faced by pharmacists in their professional environments (Makmee, 2021).

The contextual relevance of this research cannot be overstated, as it focuses specifically on the unique challenges faced by pharmacists in a rapidly industrialising economic zone. The Eastern Economic Corridor represents a microcosm of economic transformation occurring across many developing regions globally, where healthcare professionals must navigate increased patient volumes, technological advancement, cultural diversity, and evolving professional expectations. By examining well-being within this specific context, the study provides insights that extend far beyond Thailand's borders, offering a model that can be adapted for similar developing regions worldwide where healthcare systems are under pressure to modernise while maintaining quality care delivery (Office of the Eastern Economic Corridor 2024).

Perhaps most significantly, this research demonstrates systematic integration with Sustainable Development Goals (SDGs) SDG3 and SDG4, representing one of the first empirical studies to explicitly connect individual healthcare professional well-being with broader sustainable development outcomes. This integration moves beyond superficial alignment to create measurable indicators that can contribute to national SDG reporting and evidence-based policy development. By demonstrating how pharmacist well-being directly supports health workforce strengthening, universal health coverage, quality education, and lifelong learning objectives, the study provides a concrete framework for translating global development goals into actionable healthcare workforce strategies at the local level (Rowe et al., 2018).

This research strategy aligns with the United Nations Sustainable Development Goals, particularly SDG 3: Good Health and Well-being, and SDG 4: Quality Education, recognising the fundamental interconnection between healthcare professional well-being and broader sustainable development outcomes. SDG 3's commitment to ‘ensure healthy lives and promote well-being for all at all ages’ encompasses specific targets focused on strengthening health workforce capacity and achieving universal health coverage (United Nations, 2015). The well-being of healthcare professionals, including pharmacists, serves as a foundational prerequisite for achieving these ambitious targets, as their capacity to deliver consistent, high-quality healthcare services directly impacts population health outcomes across all demographic groups.

The relationship between pharmacist well-being and SDG 3 extends beyond individual professional satisfaction to encompass systemic healthcare functionality. A well-functioning pharmacy workforce contributes to essential health service delivery, medication safety protocols, and overall health system resilience – all critical components necessary for achieving SDG 3's comprehensive health agenda. This connection becomes particularly relevant in rapidly developing economic zones like the EEC, where healthcare systems must simultaneously expand access while maintaining quality standards (World Health Organization, 2016).

SDG 4's focus on ‘ensuring inclusive and equitable quality education and promoting lifelong learning opportunities for all’ maintains intrinsic connections to pharmacist well-being through multiple pathways. Target 4.4 emphasises increasing the number of people with relevant skills for employment and decent work, while Target 4.7 promotes education for sustainable development (United Nations, 2015). Pharmacists’ occupational well-being, access to professional development opportunities, and engagement in continuous learning processes directly contribute to building a skilled, adaptable health workforce capable of responding to evolving healthcare needs in the EEC's rapidly developing economic environment.

The rapid industrialisation and technological advancement characterising the EEC have fundamentally transformed the regional healthcare landscape, creating both unprecedented challenges and significant opportunities for pharmacy professionals. These transformation processes have generated increased patient volumes, introduced more complex medication regimens, expanded clinical responsibilities, and created continuous demands for professional development to maintain pace with technological innovations. Additionally, the EEC's unique multicultural environment, characterised by significant expatriate populations and diverse healthcare needs, requires pharmacists to develop cultural competency and adapt their services to meet varied cultural and linguistic requirements (Makmee, 2023).

The distinctive characteristics of the EEC setting – including swift technological progress, dynamic demographic changes, and continuous industrial structure transformation – have resulted in substantially higher workloads and increasingly intricate service requirements for healthcare professionals (Cherecheș et al., 2024). Consequently, pharmacists practicing in the region face intensified stress and pressure to maintain high service standards while simultaneously enhancing their knowledge and skills to adapt to constant environmental changes (Baines et al., 2018). These challenges, while creating potential risks to professional well-being, also present opportunities for developing innovative approaches to healthcare delivery and professional development that can serve as models for similar regions globally.

Despite the availability of comprehensive methodologies for assessing well-being among healthcare professionals in international contexts (Mayberry et al., 2022), the establishment of context-specific well-being assessment criteria for pharmacists in Thailand, particularly within the unique environment of the EEC, remains substantially underdeveloped (Khadela et al., 2021). This research gap represents a significant limitation in the ability of healthcare organisations and policymakers to understand, monitor, and support the well-being of pharmacy professionals in this critical economic development region.

The development of such assessment criteria serves multiple crucial functions, including informing evidence-based policy formulation, supporting effective human resource administration, and enabling the creation of efficient, targeted support systems for pharmacy professionals. Consistent with the policy directives established by Thailand's National Health Security Office (NHSO) and the strategic objectives outlined in the Eastern Economic Corridor Development Plan, the proposed well-being assessment criteria reinforce national priorities related to universal health coverage, sustainable workforce development, and the integration of digital health systems throughout the healthcare sector.

**Figure 1.**
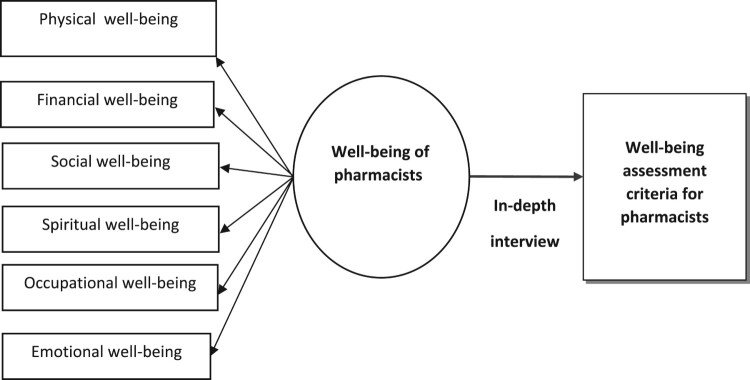
Conceptual framework of the study.

This study addresses this critical gap by examining the second-order confirmatory factor analysis of pharmacists’ well-being in the EEC context and establishing comprehensive, validated criteria for assessing pharmacy professional well-being. The research conceptualises well-being as a multidimensional construct comprising six interconnected components: physical well-being (PWb), financial well-being (FWb), social well-being (SWb), spiritual well-being (SpWb), occupational well-being (OWb), and emotional well-being (EWb). The conceptual framework of the study was shown in Figure 1.

This research provides both practical and scholarly contributions to the fields of healthcare management, occupational health psychology, and sustainable development implementation. From a practical perspective, the study identifies the essential components of pharmacists’ well-being specific to the EEC context and presents a validated set of assessment criteria that can be immediately implemented by healthcare organisations, professional associations, and policy makers. From a scholarly perspective, the research extends well-being theory to underserved professional populations in emerging economies while demonstrating innovative methodological approaches for studying professional well-being in rapidly changing economic environments.

The insights generated through this research directly inform policy development and human resource management strategies within the pharmacy profession, providing evidence-based guidance for improving the quality of work life for pharmacy professionals throughout the EEC and similar economic development regions. By establishing clear, measurable criteria for assessing pharmacist well-being, the study enables systematic monitoring of workforce conditions, identification of intervention needs, and evaluation of policy effectiveness in supporting healthcare professional development and retention.

## Literature review

### Physical well-being (PWb)

PWb is the fundamental basis of well-being. Optimal physical health allows individuals to perform their daily activities effectively. Regular exercise enhances muscular strength and cardiovascular health, diminishes the likelihood of noncommunicable diseases, and boosts energy levels for occupational and daily tasks (Zhang & Chen, 2019). Moreover, proper nutrition and sufficient sleep improve immune function and facilitate bodily recovery (Chen et al., 2020). Engaging in low-risk behaviours, including abstaining from smoking and limiting alcohol intake, can mitigate the risk of long-term health issues (Dsouza et al., 2020; Willroth et al., 2020).

### Financial well-being (FWb)

FWb is essential for establishing security and enhancing quality of life. Stable and sufficient income mitigates the stress and anxiety associated with daily expenses (Sorgente & Lanz, 2017). Effective financial planning and making emergency savings enable individuals to manage unforeseen circumstances (Riitsalu & Van Raaij, 2022). Effective debt management and planning for long-term savings are essential for achieving sustainable financial stability (She et al., 2023).

### Social well-being (SWb)

SWb significantly affects happiness and life satisfaction. Robust relationships with family, friends, and community members cultivate a sense of belonging (Veenhoven, 2015). Strong social networks provide emotional support and practical help when required (Leung et al., 2011). Engagement in social activities can improve interpersonal skills and increase self-esteem (Veenhoven, 2015).

### Spiritual well-being (SpWb)

SpWb is crucial in identifying the meaning and purpose of life. The discovery of life’s meaning serves as a source of motivation and energy for goal pursuit (Bożek et al., 2020). Inner peace, typically attained through religious or spiritual practices, helps reduce stress (Karakus et al., 2021). Compassionate and altruistic behaviours can improve life satisfaction and personal values (Frey et al., 2005).

### Occupational well-being (OWb)

OWb is significantly associated with life satisfaction and individual success. Meaningful work that aligns with individual interests enhances motivation and job engagement (Jaswal et al., 2024). A secure and nurturing workplace enhances productivity and employee satisfaction (Walsh et al., 2018). Maintaining work/life balance is essential for preventing stress and burnout (Park et al., 2023). In addition, opportunities for professional growth and advancement contribute to long-term job security and enhance self-esteem (De Neve & Ward, 2017).

### Emotional well-being (EWb)

EWb is essential for managing challenges and effectively navigating one’s life. Emotional regulation enables individuals to express and manage their emotions (Youssef-Morgan & Luthans, 2015). A positive outlook facilitates learning and enhances the capacity to identify opportunities across diverse experiences (Ryff, 1995). Adaptive capacity enables individuals to manage change and confront life challenges (Blasco-Belled & Alsinet, 2022). Recent research has demonstrated that technology-based interventions, particularly virtual reality cognitive training, can effectively enhance emotional well-being and cognitive functions through structured behavioural interventions (Makmee & Wongupparaj, 2025).

To summarise, based on a review of the relevant literature, this study identifies six fundamental components of well-being. First, PWb pertains to the preservation of health through consistent physical activity, balanced nutrition, adequate sleep, and the avoidance of hazardous behaviours, including smoking and excessive alcohol intake (Chen et al., 2020; Dsouza et al., 2020; Willroth et al., 2020; Zhang & Chen, 2019).

Second, FWb denotes the possession of financial security, adequate income, efficient financial planning, and emergency savings to manage unforeseen circumstances (Riitsalu & Van Raaij, 2022; She et al., 2023; Sorgente & Lanz, 2017).

Third, SWb entails cultivating significant relationships, maintaining a supportive social network, and engaging in community or social activities (Leung et al., 2011; Veenhoven, 2015).

Fourth, SpWb pertains to the exploration of life’s meaning, attainment of inner peace, and participation in altruistic actions that serve the welfare of others (Bożek et al., 2020; Frey et al., 2005; Karakus et al., 2021).

Fifth, OWb encompasses meaningful work, a supportive and safe work environment, work/life balance, and opportunities for career development and advancement (De Neve & Ward, 2017; Jaswal et al., 2024; Park et al., 2023; Walsh et al., 2018).

Finally, EWb includes emotional regulation, the maintenance of a positive outlook, and the capacity to adapt to changes and challenges in life (Blasco-Belled & Alsinet, 2022; Ryff, 1995; Youssef-Morgan & Luthans, 2015).

Grounded in these six components of well-being, this study proceeds with a mixed-methods approach to empirically validate and contextualise them among pharmacists in the EEC.

#### Well-being and sustainable development goals

The concept of well-being in healthcare professions has gained increased attention in the context of achieving the SDGs. Research demonstrates that healthcare worker well-being is a prerequisite for sustainable health systems (Rowe et al., 2018). SDG 3's emphasis on health workforce strengthening recognises that the well-being of health professionals directly impacts their ability to provide quality care and contribute to universal health coverage (WHO, 2016).

The six dimensions of well-being identified in this study align with multiple SDG targets. Physical well-being (PWb) directly supports SDG 3.4 (reducing non-communicable diseases) and 3.8 (universal health coverage) by ensuring pharmacists can maintain their health while providing essential services. Occupational well-being (OWb) connects to SDG 4.4 (relevant skills for employment) and 8.5 (decent work), as meaningful work and professional development opportunities are essential for sustainable healthcare delivery. Financial well-being (FWb) relates to SDG 1.3 (social protection systems) and 8.1 (economic growth), as adequate compensation and financial security enable healthcare professionals to focus on their professional responsibilities without economic distress. Social well-being (SWb) aligns with SDG 3.8's emphasis on community health and SDG 11's focus on inclusive communities. Emotional well-being (EWb) supports SDG 3.4’s mental health components, while spiritual well-being (SpWb) contributes to the holistic approach advocated in SDG 3's comprehensive health agenda.

## Methodology

This study used an explanatory sequential approach (Boun et al., 2024; Em et al., 2023; Makmee, 2021, 2023). First, quantitative research was used to investigate the six components of pharmacists’ well-being in the EEC, using questionnaires for data collection and second-order confirmatory factor analysis (CFA). Second, qualitative research was employed to gather data through in-depth interviews to augment the quantitative results. The research process comprised two phases. In Phase 1, quantitative method; the researcher analyzed and validated the second-order confirmatory factor analysis of pharmacists’ well-being in the EEC. In Phase 2, qualitative method; the researcher established assessment criteria to evaluate the well-being of pharmacists.

## Quantitative method

### Phase 1: confirmatory factor analysis of pharmacists’ well-being in the EEC

#### Population

The study’s population comprised licensed pharmacists in the EEC from three provinces, Chonburi (1,743 pharmacists), Rayong (572 pharmacists), and Chachoengsao (402 pharmacists), resulting in 2,717 individuals (Pharmacy Council of Thailand, 2024).

#### Sample and sample size determination

The sample size was established according to the recommendations of Hair et al. (2019) and Muensriphum et al. (2021), who recommended a minimum of 20 participants for each latent variable. This study, which included six components, determined the minimum required sample size to be 120 participants. To improve the reliability and generalizability of the findings, the researcher increased the sample size to 400 participants (Chiv et al., 2024; Kent et al., 2017; Prasertcharoensuk et al., 2020).

#### Sampling method

The samples were selected through simple random sampling using a drawing method to guarantee unbiased selection. The names were inscribed on individual slips of paper and drawn sequentially without replacement (Tanming et al., 2025).

#### Research instrument

This study used a questionnaire comprising two sections. Section 1 gathered demographic information, including gender, age, marital status, and educational attainment, from the respondents in a checklist format. Section 2 included 30 items aimed at assessing the six components of pharmacists’ well-being in the EEC rated on a five-point Likert-type scale from 5 = strongly agree/very high to 1 = strongly disagree/very low. The research instrument was developed and validated systematically to ensure its quality and suitability for data collection.

#### Content validity

The questionnaire was presented to five field experts to ensure the relevance, clarity, and alignment of each item with the study objectives. The Item-Objective Congruence Index (IOC) ranged from 0.67 to 1.00, which exceeded the acceptable threshold of 0.50, demonstrating that the items were well-aligned with the research objectives and content areas.

#### Reliability

The reliability of the revised questionnaire was evaluated through a pilot test administered to 30 participants who possessed characteristics similar to those of the target sample but were excluded from the main study. The Cronbach’s alpha coefficient was 0.897, indicating a high level of reliability and confirming the suitability of the instrument for the main data collection.

#### Data analysis

The sample’s characteristics were described using descriptive statistics, including frequency and percentage. The well-being of pharmacists in the EEC was assessed using descriptive statistics, including frequency, percentage, mean, and standard deviation. Second-order CFA, a statistical technique used to validate the structure of latent variables by examining the relationships between them and the underlying factors, was conducted to validate the confirmatory factor analysis of pharmacists’ well-being.

## Qualitative method

### Phase 2: assessment criteria for pharmacists’ well-being in the EEC

#### Key informants

Data were gathered from in-depth interviews focusing on the formulation of well-being assessment criteria for pharmacists in the EEC. Seven participants – selected through purposive sampling (Tanming et al., 2025), emphasising relevance, content coverage, and contextual understanding – were interviewed (Boun et al., 2024). They were selected based on the following criteria:

1. Professional experience: Currently engaged as a pharmacist in the Chonburi, Rayong, or Chachoengsao provinces for at least five years, demonstrating adequate experience to offer comprehensive insights.
2. Administrative role: Occupying a leadership or administrative position in human resource management within the pharmacy sector (e.g. Head of Pharmacy Division, Deputy Director for Support Services, or Hospital Director), allowing for contributions to policy formulation and personnel development strategies.
3. Contextual knowledge: An in-depth understanding of the EEC’s regional context, especially the economic and social transformations affecting the pharmacy profession.
4. Diverse work experience: Encompassing all six components of well-being.
5. Willingness and availability: An openness and capacity to engage in the interviews and candidly share personal experiences and perspectives.
6. Professional credibility: Recognition within the pharmacy profession and the development of robust professional networks in the EEC, thereby facilitating a comprehensive and representative viewpoint.

Participation in the study was voluntary, with written informed consent obtained before the interviews.

#### Research instrument

A semi-structured interview guide was designed to investigate the six components of pharmacists’ well-being in the EEC. Five experts in public health, pharmacy, and qualitative research evaluated its content validity to ensure its quality and relevance. The experts assessed whether the content of the questions sufficiently represented the research objectives and the components of well-being. Based on the feedback received, the interview guide was revised to enhance its clarity, coverage, and suitability for data collection.

*Qualitative Data Collection Procedures*

*Interview Protocol*

Each semi-structured interview lasted approximately 60–90 min and was conducted in a private, comfortable setting chosen by the participant. All interviews were conducted in Thai by the principal investigator, who is fluent in the language and has extensive experience in qualitative research methods.

*Interview Schedule and Environment*

Interviews were scheduled at the convenience of participants, with most taking place during non-working hours to ensure participants could speak freely without workplace pressures. The interview locations included private offices, conference rooms, or neutral venues such as quiet cafes, based on participant preference.

*Interview Process*

1. Pre-interview preparation: Participants received the interview guide 24 h before the scheduled interview to allow for reflection on the topics.
2. Opening: Each interview began with rapport-building conversation and a review of the study's purpose and participants’ rights.
3. Main interview: The semi-structured guide was followed, with probing questions used to explore responses in greater depth.
4. Closing: Participants were asked if they had additional comments and were informed about next steps.

*Recording and Documentation*

All interviews were audio-recorded using two devices to ensure backup. Field notes were taken during interviews to capture non-verbal observations and contextual information. Immediately after each interview, the researcher completed reflective memos documenting initial impressions and observations.

#### Data analysis and validation

This study employed content and inductive analyses to synthesise the key insights from the qualitative data. The analysis and validation processes by using excel programme consisted of five steps:

1. The researcher performed verbatim transcriptions of all interview recordings and carefully compared the transcripts with the original audio files to ensure data accuracy before analysis.
2. First, multiple readings of all the transcripts were conducted to understand the context and structure of the data. Second, open coding identified meaningful units that corresponded to the research questions. Third, analogous codes were classified into categories and themes. Fourth, the core elements of each theme were integrated to represent the collective experiences and viewpoints of the participants.
3. To improve data reliability, the researcher used data triangulation to assess the consistency of the information across three dimensions (Makmee, 2023; Tanming et al., 2025). Time-based triangulation involved comparing the data gathered at various time intervals. Place-based triangulation involved comparing the data collected from the participants in various locations or working for various organisations. Person-based triangulation involved comparing the data from the participants with diverse backgrounds or roles to assess the consistency of perspectives.
4. The researcher upheld neutrality during the analysis process, prioritising the participants’ voices and perspectives to minimise personal bias. Peer debriefing with subject matter experts was conducted to assess potential interpretive biases and ensure a balanced analysis critically.
5. The results were systematically organised and presented in alignment with the primary themes, supplemented by direct quotes from the participants, to underscore the significance of the findings and bolster the credibility and authenticity of the conclusions.

*Enhanced Qualitative Data Analysis*

*Step-by-Step Analysis Process*

1. Verbatim Transcription: All audio recordings were transcribed verbatim by the principal investigator within 48 h of each interview. Transcripts included not only spoken words but also significant pauses, emotional expressions, and contextual notes.
2. Data Familiarisation: The researcher read each transcript multiple times, listening to audio recordings simultaneously to ensure accuracy and gain deep familiarity with the data.
3. Initial Coding: Using an inductive approach, initial codes were generated line-by-line, staying close to participants’ language and experiences. This process was conducted manually and verified using NVivo software.
4. Code Refinement: Similar codes were grouped and refined through constant comparison. Code definitions were documented to ensure consistency.
5. Theme Development: Codes were organised into preliminary themes, which were then reviewed and refined through an iterative process involving multiple readings of the data.
6. Theme Validation: Themes were validated by checking them against the original data and ensuring they accurately represented participants’ experiences.

#### Integration of quantitative and qualitative data

The results of the second-order CFA were juxtaposed with the findings of qualitative content analysis. This integrated analysis aimed to refine and validate the six components of well-being and assessment criteria. The synthesis of both data types facilitated a comprehensive understanding of pharmacists’ well-being in the EEC and guided the creation of context-specific evidence-based assessment criteria. This integrative approach aligns with recent mixed-methods research demonstrating the effectiveness of combining behavioural and neurophysiological measures in well-being assessment (Makmee & Wongupparaj, 2025)

#### Ethical considerations

The researcher ensured ethical research was conducted by inviting and informing all the participants about the study’s objectives, procedures, and rights before data collection. The participants were explicitly informed of the voluntary nature of their involvement and their right to decline or withdraw at any time if they experienced discomfort or distress from any questions. The participants were informed that they were not required to answer any questions that made them uncomfortable and that their responses, or lack thereof, would not result in any negative consequences. All the collected data were kept confidential and used exclusively for academic purposes. Ethical approval was obtained from the Human Research Ethics, Burapha University Committee (certificate number IRB2-048/2568) on April 2, 2025.

#### SDG impact assessment framework

This study incorporates an SDG impact assessment lens to evaluate how pharmacist well-being contributes to sustainable development outcomes. The assessment criteria developed in this research are designed to support SDG monitoring and evaluation by providing measurable indicators that link individual pharmacist well-being to broader health system performance and educational outcomes.

The methodology aligns with SDG indicator frameworks by ensuring that well-being assessments can contribute to national reporting on SDG 3.c.1 (health worker density and distribution) and SDG 4.3.1 (participation in technical and vocational education). The five-level classification system enables policymakers to track progress toward SDG targets and identify areas requiring targeted interventions to achieve sustainable development outcomes.

## Results

### Quantitative phase

Altogether, 61.15% of the respondents were women. The majority of the participants were aged 30–40 years (72.00%), followed by those aged 40–50 years (16.00%), 25–30 years (11.25%), and 50–60 years (0.75%). Most were single (60.50%), followed by married (38.50%) and divorced or separated (1.00%). Regarding educational attainment, the majority held a bachelor’s degree (75.25%), followed by those with an education beyond the bachelor’s level (24.75%).

The analysis of the second-order confirmatory factor analysis of pharmacists’ well-being indicated that the standardised factor loadings (β) for all six components and the 30 items were positive and statistically significant at the 0.01 level. The standardised factor loadings of the components were organised in descending order as follows: PWb, OWb, EWb, SWb, FWb, and SpWb. The model was consistent with empirical data (*χ*^2^ = 444.99, *df* = 262, *χ*^2^/*df* = 1.70, *p* = .06, *RMSEA* = .04, *SRMR* = .06, *TLI* = .97, and *CFI* = .96) (Table 1).

**Table 1.** Results of the second-order CFA.

Variable	Standardised factor loadings								
	Physical well-being (PWb)	Financial well-being (FWb)	Social well-being (SWb)	Spiritual well-being (SpWb)	Occupational well-being (OWb)	Emotional well-being (EWb)	*SE*	*t*	*r^2^*
PWb1	.57**						.04	13.98	.33
PWb2	.69**						.03	20.42	.47
PWb3	.72**						.03	21.61	.52
PWb4	.63**						.04	16.67	.39
PWb5	.41**						.04	8.98	.16
FWb1		.37**					.05	7.60	.14
FWb2		.36**					.05	7.16	.13
FWb3		.47**					.05	8.86	.22
FWb4		.49**					.04	11.09	.24
FWb5		.63**					.05	13.70	.40
SWb1			.78**				.03	27.35	.61
SWb2			.81**				.03	32.64	.65
SWb3			.65**				.03	18.62	.42
SWb4			.60**				.03	17.42	.36
SWb5			.30**				.04	6.65	.09
SpWb1				.74**			.02	29.36	.55
SpWb2				.78**			.02	33.52	.60
SpWb3				.82**			.02	41.89	.68
SpWb4				.73**			.02	29.71	.55
SpWb5				.46**			.04	11.10	.21
OWb1					.48**		.04	11.21	.23
OWb2					.47**		.04	10.93	.22
OWb3					.75**		.03	23.43	.56
OWb4					.55**		.04	14.11	.30
OWb5					.33**		.05	7.17	.11
EWb1						.80**	.02	34.75	.64
EWb2						.79**	.02	35.26	.62
EWb3						.84**	.02	44.01	.70
EWb4						.73**	.03	28.01	.54
EWb5						.34**	.04	7.74	.12
	Latent Variables	*b*	*SE*	*t*	*R^2^*				
	PWb	.95**	.01	64.26	.68				
	FWb	.82**	.03	27.42	.77				
	SWb	.88**	.04	19.41	.43				
	SpWb	.66**	.03	19.27	.90				
	OWb	.94**	.01	69.07	.84				
	EWb	.91**	.03	33.33	.91				

Note*.* **p < .01

For PWb, the items with the highest β coefficients were adequate sleep (PWb3; *β* = 0.72), adherence to a balanced diet (PWb2; *β* = 0.69), and avoidance of alcohol consumption (PWb4; *β* = 0.63). The coefficient of determination (r²) varied between 16.00% and 52.00%.

For OWb, the items with the highest β coefficients were maintaining work/life balance (OWb3; *β* = 0.75), opportunities for professional development (OWb4; *β* = 0.55), and meaningful work that aligns with life goals (OWb1; *β* = 0.48). The r² values varied between 11.00% and 56.00%.

For EWb, the items with the highest β coefficients were effectively adapting and solving problems (EWb3; *β* = 0.84), management of stress and daily pressure (EWb1; *β* = 0.80), and optimism and the pursuit of joy in everyday life (EWb2: *β* = 0.79). The r² values varied between 12.00% and 70.00%.

For SWb, the items with the highest β coefficients were engagement in social and community activities (SWb2; *β* = 0.81), presence of close friends for emotional support (SWb1; *β* = 0.78), and perception of being a valued member of the community (SWb3; *β* = 0.65). The r² values varied between 9.00% and 65.00%.

For FWb, the items with the highest β coefficients were understanding of personal financial management (FWb5; *β* = 0.63), presence of a future savings plan (FWb4; *β* = 0.49), and maintaining emergency savings (FWb3; *β* = 0.47). The r^2^ values varied between 13.00% and 40.00%.

**Figure 2.**
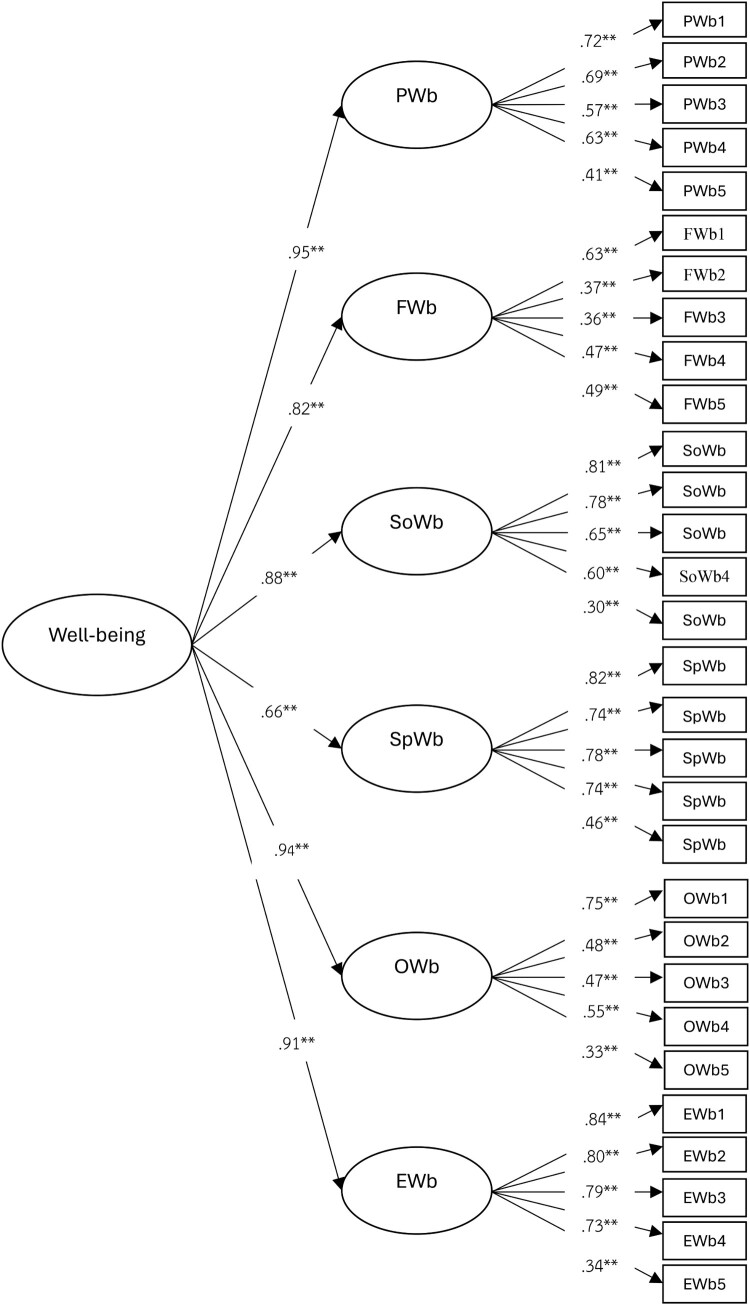
Measurement model of pharmacists’ well-being. Note: **p < .01.

For SpWb, the items with the highest β coefficients were the relationship between experiencing peace and religious practice (SpWb3; *β* = 0.82), sense of life purpose and meaning (SpWb2; *β* = 0.78), and experiencing joy and pride by assisting others (SpWb1; *β* = 0.74). The r^2^ values varied from 21.00% to 68.00%. The measurement model was shown in Figure 2.

### Qualitative phase

Table 2 shows the characteristics of the seven interviewees. The qualitative findings closely aligned with the quantitative results, underscoring the significance of each component, particularly those with the highest standardised factor loadings shown above.

**Table 2.** Personal characteristics of the interviewees.

Characteristic	Frequency *(n)*	Percentage
Gender		
Male	2	28.57
Female	3	42.86
Gender Diverse	2	28.57
Years of Work Experience		
5–10 years	2	28.57
10–15 years	2	28.57
More than 15 years	3	42.86
Current Position		
Head of Pharmacy Division	1	14.29
Chief of Pharmaceutical Services	2	28.57
Deputy Director (Support Services)	4	57.14

For PWb, the participants highlighted the significance of proactive health maintenance. Their daily practices demonstrated significant involvement in health-promoting behaviours, as evidenced by the following quotes:

Preventive self-care involves taking proactive measures to maintain good health and overall well-being.

I engage in physical exercise consistently, a minimum of 3–4 times weekly.

I ensure the daily consumption of six to eight glasses of water.

I allocate my time to intervals; after two hours of work, I take a 10- to 15-minute break to stretch.

Challenges related to health:

Pharmacy counters and chairs lack comfort. I frequently encounter shoulder and lower back discomfort during work hours.

Prolonged standing results in muscle and joint discomfort, including back and leg pain.

For OWb, the interviewees articulated concerns about workload and professional recognition.

High patient volumes and inadequate staffing contribute to decreased job satisfaction. This was convenient for daily use.

My job satisfaction is average due to the substantial workload.

Recognition as a medication expert, rather than merely a dispenser, serves as a significant motivator.

For EWb, the pharmacists developed various stress management techniques and methods for emotional self-regulation.

I engage in viewing series and online shopping as means of relaxation.

I engage in deep breathing exercises or brief mindfulness meditations throughout the day.

I participate in hobbies to distinguish work from my personal life.

Divergent viewpoints among colleagues influence my emotional state.

Individuals and bureaucratic systems serve as the primary sources of emotional stress.

For SWb, workplace relationships have a substantial impact on the well-being of the pharmacists. Social support and connection are critical components of well-being.

Support and recognition from the system and colleagues is essential.

Colleagues and patient load significantly influence workplace satisfaction.

Social balance in work/life integration:

I distinguish between professional and personal time by using my free time to engage with family and friends.

I allocate time to familial and personal engagement.

For FWb, financial stability, while not extensively addressed, remains pertinent. Income security and benefits are fundamental to well-being.

It is essential to balance work, finances, health, and personal life.

For SpWb, the participants’ sense of purpose in their professional roles was indirectly indicated.

Observing positive outcomes from our efforts, such as enhanced patient health and expressions of gratitude, provides a sense of satisfaction.

The integration into a healthcare team contributes to increased happiness and motivation.

Based on the findings from both the quantitative and the qualitative phases, the researcher developed well-being assessment criteria for pharmacists in the EEC, which were validated and refined through additional expert interviews. Each indicator in the criteria received one point when evidence of implementation was present. Each indicator of one of the six components received one point if all the indicators were satisfied. The final scoring system integrated the weighted values derived from the standardised factor loadings (β) obtained through the second-order CFA to guarantee that the assessment criteria captured both the statistical significance of each element (derived from the quantitative phase) and its practical relevance and applicability (informed by the qualitative phase). The maximum score for each component varied based on the number of indicators and the relative weights assigned to each factor (see Table 3).

**Table 3.** Calculation of the scores for pharmacists’ well-being assessment criteria.

Well-Being Component	Scoring Formula Based on the Standardised Loadings (β)	Maximum Score
Physical Well-being (PWb)	((.57 × 1) + (.69 × 1) + (.72 × 1) + (.63 × 1)) + (.41 × 1) x .95	2.87
Occupational Well-being (OWb)	((.48 × 1) + (.47 × 1) + (.75 × 1) + (.55 × 1)) + (.33 × 1) x .94	2.43
Emotional and Psychological Well-being (EWb)	((.80 × 1) + (.79 × 1) + (.84 × 1) + (.73 × 1)) + (.34 × 1) x .91	3.19
Social Well-being (SWb)	((.78 × 1) + (.81 × 1) + (.65 × 1) + (.60 × 1)) + (.30 × 1) x .88	2.76
Financial Well-being (FWb)	((.37 × 1) + (.36 × 1) + (.47 × 1) + (.49 × 1)) + (.63 × 1) x .82	1.90
Spiritual Well-being (SpWb)	((.74 × 1) + (.78 × 1) + (.82 × 1) + (.73 × 1)) + (.46 × 1) x .66	2.33
	Total Maximum Score	15.48

Table 3 presents the well-being assessment scores for the pharmacists in the EEC, according to the criteria established by the researcher. The width of each scoring-level class interval is determined as follows:

$$\begin{array}{rl} \mathrm{The}\;\mathrm{width}\;\mathrm{of}\;\mathrm{the}\;\mathrm{class}\;\mathrm{interval}\;\mathrm{for}\;\mathrm{the}\;\mathrm{assessment}\;\mathrm{score}\;\mathrm{levels} & =\frac{\left(\mathrm{Maximum}\;\mathrm{score}\text{–}\mathrm{Minimum}\;\mathrm{Score}\right)}{\mathrm{Interval}}\\ & =\frac{\left(15.48\text{–}0\right)}{5}\\ & =3.10 \end{array}$$

The overall score was used to classify pharmacists’ well-being into five levels, as detailed below:

Level 1 (Needs Improvement): Score between 0.00 and 3.10 indicates that pharmacists’ well-being is critically low and necessitates substantial enhancement.

Level 2 (Fair): Score between 3.11 and 6.20 indicates that pharmacists’ well-being is at a foundational level, suggesting potential for improvement.

Level 3 (Good): Score between 6.21 and 9.30 indicates that pharmacists exhibit a moderate level of well-being.

Level 4 (Very Good): Score between 9.31 and 12.40 indicates that the pharmacists’ well-being is high, reflecting favourable conditions.

Level 5 (Excellent): Score between 12.41 and 15.49 indicates that pharmacists experienced a significantly high degree of well-being in all six components.

## Discussion

### Model fit

The confirmatory factor analysis of the pharmacist well-being measurement model revealed that the model was consistent with empirical data, as evidenced by *χ*^2^ = 444.99, *df* = 262, *χ*^2^/*df* = 1.70, *p* = .06, *RMSEA* = .04, *SRMR* = .06, *TLI* = .97, and *CFI* = .96, which makes this analytical model acceptable. Additionally, the test results showed that χ² did not differ significantly from zero, meaning the hypothesis that the measurement model has construct validity was accepted. This is consistent with the χ^2^/*df* analysis results showing a value less than 2, the *RMSEA* index value less than .05, *SRMR* value less than .08, and *TLI* and *CFI* values close to 1 (Hair et al., 2019; Kelloway, 2014).

### Prioritisation of the well-being components

#### PWb: a fundamental component of overall well-being

PWb exhibited the highest factor loading (*β* = 0.95), thereby affirming its essential role in overall well-being. This finding aligns with that of Zhang and Chen (2019), who highlighted the foundational role of physical health to overall well-being. The pharmacists’ focus on exercise, adequate sleep, and balanced nutrition demonstrates a proactive strategy for addressing the physical challenges associated with extended periods of standing. The qualitative findings indicated prevalent issues resulting from inadequate work environments, including back and shoulder pain, corroborating the conclusions of Chen et al. (2020), who suggested that physical health positively influences happiness and work performance.

#### OWb: the issue of professional recognition

OWb was ranked second (*β* = 0.94). The pharmacists indicated a preference for recognition as experts in medication rather than simply as dispensers, a notion strongly corroborated by the qualitative data. This is consistent with Alzarea et al. (2022), who observed an increasing involvement of pharmacists in supply chain management, clinical consultation, and the promotion of rational drug use. Increasing workload and patient volume present challenges in maintaining service quality amid economic pressures, as analyzed by Baines et al. (2018) in their examination of modern pharmacy practice complexities. This emphasis on professional recognition aligns with research showing that pharmacists’ learning approaches and professional development orientation significantly impact their career satisfaction (Phanudulkitti et al., 2018). The desire for recognition as medication experts rather than dispensers reflects a continuation of the deep learning approach that supports professional identity formation.

#### EWb: strategies for managing stress

EWb ranked as the third most significant component (*β* = 0.91). The pharmacists used various stress management techniques, including leisure activities such as watching series and online shopping as well as performing mindfulness and breathing exercises. These findings are consistent with those of Youssef-Morgan and Luthans (2015), who highlighted the importance of emotional regulation and positive attitudes toward workplace well-being. The capacity of the pharmacists to adapt and devise solutions exemplifies psychological resilience, a crucial attribute of high-responsiveness healthcare professionals.

#### SWb: the influence of support networks

The results for SWb (*β* = 0.88) underscored the significance of colleague support and organisational recognition. This aligns with the findings of Leung et al. (2011), who indicated that robust social networks offer emotional support and practical assistance. The emphasis of pharmacists on delineating professional responsibilities from personal time and prioritising quality interactions with family and friends demonstrates a deliberate attempt to achieve life balance, aligning with Veenhoven’s (2015) perspective of the significance of meaningful relationships for overall happiness and life satisfaction.

#### FWb: stability as a fundamental basis

FWb ranked fifth (*β* = 0.82). The participants indicated that balancing work, finances, health, and personal life significantly contributes to well-being, although this topic was not extensively discussed. This corroborates the findings of Sorgente and Lanz (2017), who noted that an adequate and stable income alleviates the stress associated with daily expenses. Personal financial management and future savings are significant concerns for long-term financial security.

#### Spwb: significance and objectives in employment

Despite being the lowest-ranked component (β = 0.66), SpWb contributed significantly. The pharmacists demonstrated commitment to patient care, reflecting an intrinsic motivation that transcends personal gain. The findings align with Bożek et al. (2020), who identified that finding meaning in life offers both energy and direction. Demonstrating compassion and forgiveness in challenging situations indicates substantial personal development, which is crucial for addressing emotional challenges inherent in the profession.

### Integration of the quantitative and qualitative findings

The application of a mixed-methods explanatory sequential approach yielded a more thorough and nuanced understanding of the pharmacists’ well-being than a single-method design. This is consistent with the view of Em et al. (2023), who highlighted the advantages of combining various data sources (Makmee, 2023). The quantitative results demonstrating that PWb exhibited the highest factor loading (*β* = 0.95) were strongly supported by the qualitative data. This convergence enhanced the credibility and reliability of prioritising the components of well-being. The interviews indicated that the pharmacists participated in organised health practices, including exercising regularly (at least 3–4 times per week) and dividing work into intervals (e.g. working for 2 h and taking a 10–15 min break).

The second-highest score of OWb (*β* = 0.94) was corroborated by the qualitative feedback from the pharmacists, who expressed a desire for professional recognition (‘not being seen as just a dispenser, but as a true medication expert’) and reported dissatisfaction stemming from workload (‘I feel less happy because there are too many patients and too few staff’).

For PWb, the interviews revealed that the physical strain suffered was associated with job demands: ‘Standing for long periods causes muscle and bone pain such as back and leg discomfort’ and ‘dispensing counters and chairs are uncomfortable.’ This analysis elucidates the importance of physical healthcare for pharmacists.

The lower factor loading for SpWb (*β* = 0.66) observed in the quantitative analysis was verified by the qualitative data, as evident in statements such as ‘seeing positive outcomes from work such as improving patients,’ which suggests a profound sense of meaning and purpose in their professional roles.

#### Contribution to sustainable development goals

The findings of this study provide substantial evidence for how pharmacist well-being contributes to achieving SDGs 3 and 4. The prioritisation of physical well-being (*β* = 0.95) aligns with SDG 3's emphasis on ensuring healthy lives, as pharmacists who maintain their physical health are better positioned to deliver consistent, quality healthcare services. This finding supports SDG 3.c, which calls for substantially increasing health workforce recruitment, development, training, and retention.

The high loading for occupational well-being (*β* = 0.94) directly supports SDG 4's focus on quality education and lifelong learning. Pharmacists’ emphasis on professional recognition and meaningful work reflects the importance of continuous professional development, aligning with SDG 4.4's target of increasing the number of people with relevant skills for employment. The qualitative findings revealing desires for recognition as ‘medication experts rather than dispensers’ demonstrate the profession's evolution toward higher-skilled, knowledge-based practice – a key component of SDG 4's vision for education and skills development.

The study’s focus on the Eastern Economic Corridor provides insights particularly relevant to SDG implementation in middle-income countries undergoing rapid economic transformation. The challenges identified – including workload pressures, infrastructure limitations, and professional recognition issues – reflect common barriers to achieving SDGs 3 and 4 in developing economic zones globally.

### Development of well-being assessment criteria

The assessment criteria were formulated using a weighted scoring method, informed by the standardised factor loadings obtained from the quantitative analysis. The priority order of each component was then established from the qualitative data. This scoring system indicated the relative importance of each component as perceived by pharmacists in the EEC. The design of the five-level classification system adhered to the principles of continuous quality improvement in healthcare, enabling organisations to track progress and set objectives.

Although EWb achieved the highest total score, this did not indicate that it was the most critical component. The score was derived from its substantial number of indicators and robust factor loadings. Conversely, PWb exhibited the highest coefficient (*β* = 0.95), underscoring its statistical significance in elucidating overall well-being.

### Theoretical contributions and practical applications

This study builds on the well-being theories established by Ryff (1995) and Diener et al. (2018) by situating them within the context of the pharmacy profession. This study corroborates the results of a recent systematic literature review and meta-analysis that showed high confidence that lack of professional recognition can lead to workplace alienation among pharmacists globally (Forsyth et al., 2025). This emphasises the variability in the significance of the well-being components based on professional roles and work contexts. In contrast to other professionals who prioritise EWb or SWb, PWb is paramount to pharmacists because of the physical demands inherent to their roles.

The creation of localised well-being assessment criteria demonstrated a context-specific model designed for the EEC. This analysis considers regional factors, including economic transformation, infrastructure development, and increasing demand for healthcare services, in line with the findings of Makmee (2023) on the effects of regional development. These criteria provide a systematic instrument for assessing pharmacists’ well-being to predict trends and identify areas for enhancement. The rating system improves usability and facilitates effective communication between stakeholders.

The assessment results can inform the design of targeted well-being programmes. For instance, if PWb scores are low, an organisation may implement workplace exercise programmes, improve chairs and workstations, or introduce health promotion initiatives, all of which are supported by the qualitative feedback from the participants. Hence, these criteria can guide human resource policies encompassing budget allocation for workplace enhancement, suitable pharmacist-to-patient ratios, and career advancement strategies. These measures respond to the critical issues identified in the qualitative findings and enhance sustainable workforce well-being.

#### SDG implementation implications

The well-being assessment criteria developed in this study offer practical tools for SDG implementation at the healthcare system level. The five-level classification system provides a framework for monitoring progress toward SDG targets by enabling systematic assessment of health workforce well-being as a foundation for health system strengthening.

For SDG 3 implementation, the criteria support Target 3.8 (universal health coverage) by ensuring pharmacists have the well-being foundation necessary to deliver essential health services. The emphasis on physical and occupational well-being directly contributes to Target 3.c (health workforce strengthening) by providing measurable indicators for workforce capacity and sustainability.

For SDG 4 implementation, the occupational well-being component supports Target 4.4 (relevant skills for employment) by emphasising professional development and meaningful work. The assessment criteria can inform educational policies and professional development programmes that align with SDG 4's vision of quality, inclusive education.

### Policy and practice recommendations

This research highlights the need to enhance the design of dispensing counters and chairs in pharmacies and provide suitable lighting and ventilation systems, consistent with the findings of Walsh et al. (2018), who highlighted the significance of physical work environments in improving job performance and well-being. Organisations and leaders must also actively advocate the professional role of pharmacists as experts in medication management by providing opportunities for involvement in clinical decision making, ongoing professional development, and public awareness initiatives. This aligns with the findings of Price et al. (2023) on the enhancement of pharmacists’ roles within healthcare systems.

The interviews highlighted concerns about the disparity between patient volume and pharmacist staffing. Organisations should thus enhance their workforce capacity, implement flexible scheduling, and ensure equitable task distribution to mitigate stress and prevent burnout. The recommendations align with the findings of Cherecheș et al. (2024) on the challenges in managing pharmacists’ workload. Based on the findings of this study, the following policy recommendations are proposed:

1. The Ministry of Public Health and the Pharmacy Council of Thailand should establish policies regarding workplace standards in pharmacy practice, including appropriate counters, chairs, lighting, and ventilation systems, in order to prevent health problems caused by prolonged standing and unsuitable equipment.
2. The Pharmacy Council of Thailand and hospitals should formulate policies to strengthen the professional role of pharmacists by creating clear career advancement pathways and supporting continuous professional development.
3. The Department of Medical Services and the Office of the Civil Service Commission (OCSC) should enhance workforce management by determining an appropriate pharmacist-to-patient ratio and ensuring equitable workload distribution, thereby reducing stress and overwork among pharmacists.
4. The National Health Security Office (NHSO) and the Department of Mental Health should develop policies to establish a comprehensive system for assessing and promoting pharmacists’ well-being, encompassing physical, occupational, emotional, social, financial, and spiritual dimensions, to enable systematic monitoring and the implementation of targeted support measures.
5. Government agencies, educational institutions, and healthcare organisations should collaborate to create integrated approaches to pharmacist well-being that support both health system strengthening (SDG 3) and educational quality (SDG 4).

### Future research directions

Future research could broaden its geographic focus to compare well-being across other regions in Thailand. Studies could also incorporate multilevel analyses to examine the impact of organisational- and policy-level factors on pharmacists’ well-being. A longitudinal study design would elucidate the evolution of well-being over time and identify the factors influencing these changes. Additional research directions include: (1) intervention studies to test the effectiveness of well-being programmes based on these criteria, (2) comparative studies across different healthcare professions, and (3) economic analyses of the cost-effectiveness of well-being interventions in pharmacy practice.

## Conclusion

This mixed-methods study successfully validated a six-component well-being framework for pharmacists in Thailand's Eastern Economic Corridor. The confirmatory factor analysis revealed Physical Well-being as the most critical component (*β* = 0.95), followed by Occupational Well-being (*β* = 0.94) and Emotional Well-being (*β* = 0.91). The qualitative findings strongly corroborated these quantitative results, with pharmacists emphasising the fundamental importance of physical health maintenance, professional recognition, and effective stress management strategies.

Theoretically, this research extends existing well-being theories by demonstrating context-specific variations in component prioritisation within healthcare professions. Unlike other professional groups that typically prioritise emotional or social dimensions, pharmacists’ emphasis on physical well-being reflects the unique occupational demands of their role. Practically, the study provides evidence-based assessment criteria that enable systematic evaluation and monitoring of pharmacist well-being across multiple dimensions.

The validated five-level classification system (ranging from ‘Needs Improvement’ to ‘Excellent’) offers healthcare organisations, policymakers, and professional associations a standardised tool for workforce management and targeted intervention design. These criteria can inform workplace improvements, staffing policies, professional development programmes, and comprehensive wellness initiatives that address the evolving challenges facing pharmacy professionals in Thailand's rapidly developing economic zones. The framework establishes a foundation for evidence-based approaches to enhancing both individual pharmacist well-being and overall healthcare system performance.

This study contributes to the global effort toward achieving Sustainable Development Goals 3 and 4 by providing evidence-based tools for assessing and enhancing pharmacist well-being. The validated framework demonstrates how individual professional well-being connects to broader sustainable development outcomes, offering policymakers and healthcare leaders practical approaches for workforce strengthening that support both quality health service delivery and continuous professional development.

The research provides a replicable model for other middle-income countries seeking to strengthen their health workforce while pursuing rapid economic development. By aligning pharmacist well-being assessment with SDG frameworks, this study contributes to the evidence base for sustainable health system development and offers pathways for integrating workforce well-being into national SDG implementation strategies.

Future research should explore the quantitative relationships between pharmacist well-being scores and specific SDG indicators, enabling more precise measurement of how professional well-being contributes to sustainable development outcomes. Such research would strengthen the evidence base for investing in healthcare workforce well-being as a strategy for achieving the 2030 Agenda for Sustainable Development.
